# From Knock-Out Phenotype to Three-Dimensional Structure of a Promising Antibiotic Target from *Streptococcus pneumoniae*

**DOI:** 10.1371/journal.pone.0083419

**Published:** 2013-12-13

**Authors:** Con Dogovski, Michael A. Gorman, Natalia E. Ketaren, Judy Praszkier, Leanne M. Zammit, Haydyn D. Mertens, Gary Bryant, Ji Yang, Michael D. W. Griffin, F. Grant Pearce, Juliet A. Gerrard, Geoffrey B. Jameson, Michael W. Parker, Roy M. Robins-Browne, Matthew A. Perugini

**Affiliations:** 1 Department of Biochemistry, La Trobe Institute for Molecular Science, La Trobe University, Melbourne, Victoria, Australia; 2 Department of Biochemistry and Molecular Biology, Bio21 Molecular Science and Biotechnology Institute, University of Melbourne, Victoria, Australia; 3 St Vincent's Institute of Medical Research, Fitzroy, Victoria, Australia; 4 Department of Microbiology & Immunology, University of Melbourne, Victoria, Australia; 5 Australian Synchrotron, Clayton, Victoria, Australia; 6 School of Applied Sciences, RMIT University, Melbourne, Victoria, Australia; 7 Biomolecular Interaction Centre and School of Biological Sciences, University of Canterbury, Christchurch, New Zealand; 8 Callaghan Innovation, Lower Hutt, New Zealand; 9 Centre for Structural Biology, Institute of Fundamental Sciences, Massey University, Palmerston North, New Zealand; University of Edinburgh, United Kingdom

## Abstract

Given the rise in drug-resistant *Streptococcus pneumoniae*, there is an urgent need to discover new antimicrobials targeting this pathogen and an equally urgent need to characterize new drug targets. A promising antibiotic target is dihydrodipicolinate synthase (DHDPS), which catalyzes the rate-limiting step in lysine biosynthesis. In this study, we firstly show by gene knock out studies that *S. pneumoniae* (*sp*) lacking the DHDPS gene is unable to grow unless supplemented with lysine-rich media. We subsequently set out to characterize the structure, function and stability of the enzyme drug target. Our studies show that *sp*-DHDPS is folded and active with a *k*_cat_ = 22 s^-1^, *K*_M_^*PYR*^ = 2.55 ± 0.05 mM and *K*_M_^*ASA*^ = 0.044 ± 0.003 mM. Thermal denaturation experiments demonstrate *sp*-DHDPS exhibits an apparent melting temperature (*T*_M_^app^) of 72 °C, which is significantly greater than *Escherichia coli* DHDPS (*Ec*-DHDPS) (*T*_M_^app^ = 59 °C). Sedimentation studies show that *sp*-DHDPS exists in a dimer-tetramer equilibrium with a *K*_D_^4→2^ = 1.7 nM, which is considerably tighter than its *E. coli* ortholog (*K*_D_^4→2^ = 76 nM). To further characterize the structure of the enzyme and probe its enhanced stability, we solved the high resolution (1.9 Å) crystal structure of *sp*-DHDPS (PDB ID 3VFL). The enzyme is tetrameric in the crystal state, consistent with biophysical measurements in solution. Although the *sp*-DHDPS and *Ec*-DHDPS active sites are almost identical, the tetramerization interface of the s. *pneumoniae* enzyme is significantly different in composition and has greater buried surface area (800 Å^2^) compared to its *E. coli* counterpart (500 Å^2^). This larger interface area is consistent with our solution studies demonstrating that *sp*-DHDPS is considerably more thermally and thermodynamically stable than *Ec*-DHDPS. Our study describe for the first time the knock-out phenotype, solution properties, stability and crystal structure of DHDPS from *S. pneumoniae*, a promising antimicrobial target.

## Introduction

*Streptococcus pneumoniae* is a Gram-positive bacterium and human commensal inhabiting the upper respiratory tract [1]. The organism often causes pneumonia in children, the elderly and immunocompromized, and if left untreated can result in death [2]. Pneumonia accounts for the death of approximately 1 million children per annum under the age of 5, making this disease the leading cause of childhood mortality worldwide [2,3]. In recent years *S. pneumoniae* has received considerable attention due to the emergence of multi-drug resistant strains, commonly referred to as drug-resistant Streptococcus pneumoniae (DRSP) [3,4]. Standard antimicrobial treatment options, such as the β-lactam and macrolide antibiotics, are becoming less effective due to the rise in DRSP [4-7]. This is in part due to the promiscuous nature of *S. pneumoniae* in acquiring genetic resistance elements from other bacteria, accompanied by selective pressure as a result of high antibiotic usage [8,9]. Alongside the use of antibiotic agents to combat infection, vaccination is available as a preventative measure [10]; however, current pneumococcal vaccines do not offer protection against all infectious strains. Thus there is an urgent need to discover new therapeutics targeting appropriate biomolecules from *S. pneumoniae*. 

**Figure 1:**
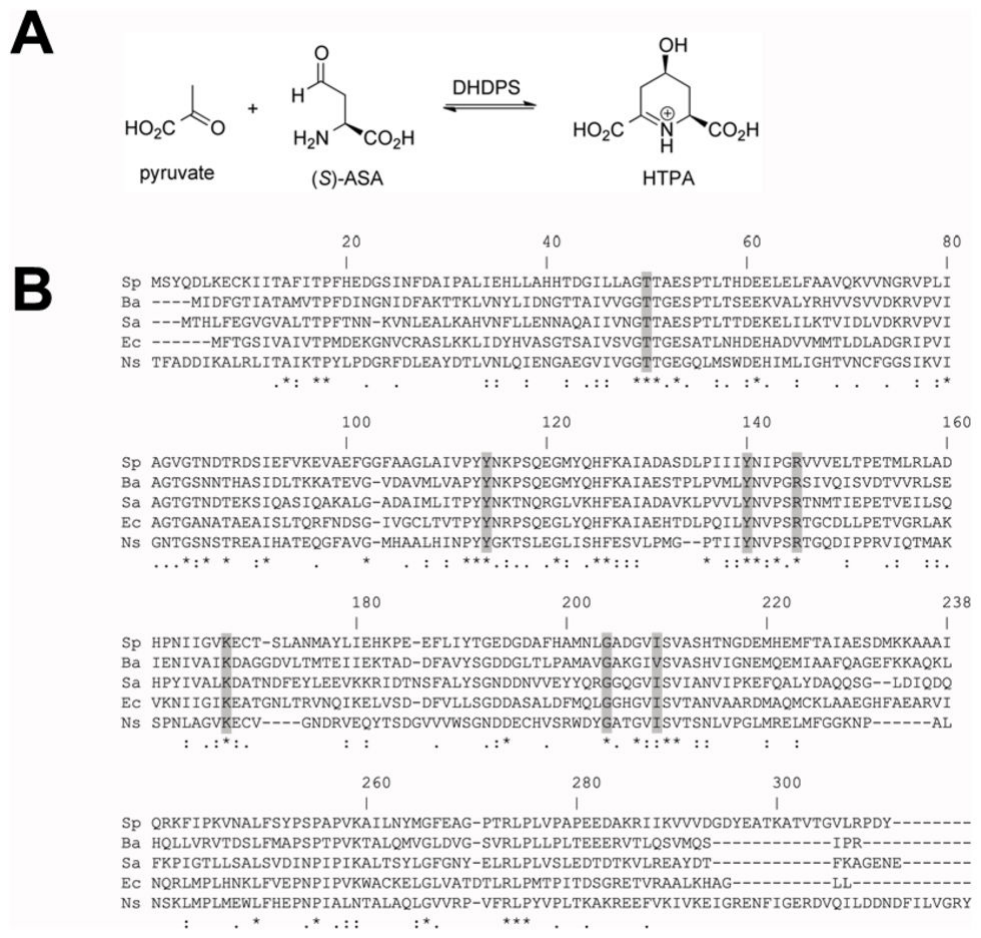
Enzymatic reaction and multiple sequence alignment of DHDPS. (A) Condensation reaction catalyzed by DHDPS. (B) Multiple sequence alignment of DHDPS sequences from bacteria, namely *S. pneumoniae* (Sp), *B. anthracis* (Ba), *S.* aureus (Sa), and *E. coli* (Ec), and also the plant species *N. sylvestris* (Ns). Conserved active-site residues are shaded grey.

A promising antimicrobial target is the enzyme dihydrodipicolinate synthase (DHDPS), which catalyzes the first committed step in the lysine biosynthetic pathway of bacteria, namely the condensation of pyruvate and (*S*)-aspartate semialdehyde [(*S*)-ASA] to form the product, hydroxytetrahydrodipicolinic acid (HTPA) (Figure 1A) [11-14]. Humans do not synthesize lysine *de novo* and thus acquire this essential amino acid from dietary sources; whereas bacteria, such as *S. pneumoniae*, synthesize lysine *de novo* for both protein and cell-wall synthesis [11-14]. The absence of a lysine biosynthetic pathway in humans and the fact that lysine is a fundamental building block of proteins and peptidoglycan in bacteria, highlights the potential for targeting the enzymatic machinery involved in this pathway for novel antibiotic discovery [11-15].

To date, almost all characterized DHDPS enzymes, excluding notable exceptions from *Staphylococcus aureus* [16,17] and *Pseudomonas aeruginosa* [18,19], adopt a homotetrameric structure [20-36]. Each monomeric unit folds to form a TIM-barrel, or (β/α)_8_ topology, which subsequently self-associates to form a tetramer or dimer of ‘tight’ dimers [20-36]. Tetramerization of DHDPS is shown to be important for stabilizing conformational dynamics of the ‘tight’ dimer interface where the key active-site residues are located [26,27,36]. These include K161 (*E. coli* numbering), which forms a Schiff base with the first substrate to bind the enzyme (i.e. pyruvate), and a catalytic triad comprised of Y107, T44 and Y133, which are strongly conserved in all DHDPS enzymes characterized to date [25,31] including *S. pneumoniae* (Figure 1B). 

Given the clinical importance of *S. pneumoniae* and the rise in multi-drug resistance in this Gram-positive pathogen, the aims of this study were to (i) determine the phenotype of a DHDPS gene knock mutant of *S., pneumoniae*; (ii) characterize the solution properties, stability and catalytic activity of *Sp*-DHDPS; and (iii) determine the high-resolution crystal structure of the enzyme to afford structure-based drug design strategies in future studies.

## Materials and Methods

### Bacterial strains, media, and chemicals

*Escherichia coli* K-12 TOP10 cells (Invitrogen, Carlsbad, CA), grown in Luria-Bertani (LB) medium, were used for preparation of plasmid DNA. *Escherichia coli* BL21(DE3) strain grown in LB medium was used for recombinant protein expression. *S. pneumoniae* 774A, isolated from CSF of a child with meningitis [37] was grown routinely in Brain Heart Infusion (BHI) broth or on Horse Blood Agar (HBA) plates, at 37°C in an atmosphere of 5% CO_2_. The chemically defined medium with (CDM^+^), or without (CDM^-^), (*S*)-lysine (200 µg ml^-1^) was that described by van de Rijn and Kessler [38], supplemented with choline chloride (5 µg ml^-1^), asparagine (50 µg ml^-1^), and sodium pyruvate 250 µg ml^-1^) as described by Moscoso et al. [1]. Ampicillin was used at a final concentration of 50 µg ml^-1^, chloramphenicol at 10 µg ml^-1^, erythromycin at 0.1 µg ml^-1^, and 5-bromo-4-chloro-3-indolyl-β-D-galactopyranoside (X-Gal) at 25 µg ml^-1^. For growth studies, cells from freshly grown single colonies on HBA plates were inoculated into 3 ml of CDM^+^ and 3 ml CDM^-^. Cultures were incubated statically for 16 hours in an atmosphere of 5% CO_2_ at 37°C. Growth was assessed by monitoring the optical density (Absorbance) of duplicate cultures on an hourly basis using a Klett-Summerson photoelectric colorimeter (filter no. 54, spectral range 520-580 nm).

### Recombinant DNA techniques

Routine DNA manipulations were performed using standard techniques [39]. Plasmid DNA was purified using Wizard plus SV DNA purification system (Promega, Madison, WI). PCR amplifications were performed with Phusion High-Fidelity DNA Polymerase (Finnzymes Oy, Finland) or high-fidelity Platinum Taq DNA polymerase (Invitrogen, Carlsbad, CA). DNA derived from PCR reactions was purified using the UltraClean PCR Clean-up Kit (Mo Bio Laboratories, Inc.). Oligonucleotides were purchased from GeneWorks Pty. Ltd. (Hindmarsh, South Australia, Australia).

### Construction of the S. pneumoniae 447A ΔdapA mutant strain

Overlapping extension PCR [40] was used to generate a DNA fragment carrying the erythromycin resistance (Em^R^) gene flanked by regions up and downstream of *S. pneumoniae* 447A *dapA*. Briefly, primer pairs dap.F/dapERM.R and dapERM.F/dap.R (Table 1) were used to amplify DNA flanking the region to be deleted from the chromosome of *S. pneumoniae* 447A, and primers pVA838.F/pVA838.R (Table 1) were used to amplify the Em^R^ gene from plasmid pVA838 [41]. The products of these three PCR reactions (100 ng each) served as template in overlapping extension PCR using primers dap.F/dap.R (Table 1) to generate a linear construct, which was cloned into pGEM-T Easy (Promega, Madison,WI), introduced into *E. coli* K-12 TOP10 cells and confirmed by sequencing. The pGEM-T Easy construct was used as a template in a PCR with primers dap.F/dap.R, to amplify the linear allelic replacement DNA fragment, which was introduced into *S. pneumoniae* 447A by transformation. The *ΔdapA* mutation was confirmed by PCR using primer pairs in which one primer flanked the targeted region and the other primed within the Em^R^ gene (OCD52/dapERM.R and OCD53/dapERM.F). The PCR products were sequenced using primers OCD52 and OCD53 (Table 1).

**Table 1:** Sequences of primers employed in the S. *pneumoniae dapA* knock out experiments.

**Primer**	**Sequence**
pVA838.F	CACAAGTGATTTGTGATTGTTG
pVA838.R	GCGCTTAGTGGGAATTTGTAC
dapERM.F	GTACAAATTCCCACTAAGCGCGTCGTAGATGGCGACTACGAAGC
dapERM.R	CAACAATCACAAATCACTTGTGCAATCAAGGCTGGAATAGCATC
dap.F	CGAAGAGATGAAGATGACCAAGG
dap.R	GAATCAACAACCTCTTCTTTGAAAATGC
OCD52	CACGTGATTTGCATGCGGAA
OCD53	ATCGGTGTTGAGCGTTCGAA

### Transformation of *S. pneumoniae* 447A

Bacteria were grown in c-CAT medium (1% w/v Casamino acids, 0.5% w/v Tryptone, 0.5% w/v NaCl, 1% w/v Yeast Extract, 16 mM K_2_HPO_4_, 0.2 % w/v glucose, 15 μg ml^-1^ glutamine) at 37°C to OD_600_ of 0.25-0.30. Cells were diluted 1/10 in 10 ml CTM medium (c-CAT containing 0.2% BSA and 1 mM CaCl_2_), grown at 37°C to OD_600_ of 0.10, collected by centrifugation and resuspended in 1 ml of 15% v/v glycerol prepared in CTM adjusted to pH 7.8. 100 μl aliquots of cell suspension were stored at -80°C until required. For transformation, 100 µl of cells were thawed on ice, 1 ml of CTM-pH 7.8 and 100 ng of synthetic competence-stimulating peptide 1 (CSP-1) [42] were added and cells incubated at 37°C for 13 min. DNA was added and cells were incubated at 32°C for 35 min then incubation continued for 3 hours at 37°C. Transformation mixture was plated out on HBA containing 0.1 µg ml^-1^ erythromycin and incubated overnight at 37°C in an atmosphere of 5% CO_2_.

### Expression and purification of *Sp*-DHDPS

Recombinant *Sp*-DHDPS was expressed, purified and assessed by SDS-PAGE and mass spectrometry to be >95% homogeneous as described previously [43].

### Coupled kinetics assay

Kinetic studies were conducted using the coupled-assay method as previously described [16,36,44,45]. Data were collected on a Cary UV-Vis spectrophotometer (Varian) connected to a Peltier cell to maintain a constant temperature of 30°C. Assays were performed in 1.5 ml semi-micro acrylic cuvettes with a path length of 10 mm. (S)-ASA was synthesized according to the methods of Roberts et al. [46]. A standard assay contained 20 nM of DHDPS, 250 mM HEPES pH 8.0, 0.2 mM NADPH, varied concentrations of pyruvate and (*S*)-ASA, 75 µg ml^-1^ of DHDPR (purified from *E. coli*) in a final volume of 0.8 ml. Cuvettes were pre-incubated at 30°C for 8 min before the reaction was initiated *via* the addition of (*S*)-ASA. Rates were determined from the initial linear portion of the data collected. The background degradation of NADPH was factored into rate calculations and each data point was measured in triplicate within a <10% error margin. 

### Circular dichroism (CD) spectroscopy

CD spectroscopy experiments were performed on an Aviv Model 420SF CD spectrometer using a 1.0 nm bandwidth. CD spectra of *Sp*-DHDPS were recorded at a protein concentration of 4.5 µM solubilized in 20 mM Tris-HCl pH 8.0 and 150 mM NaCl. Wavelength scans spanning 195-240 nm were measured using a step size of 0.5 nm with a 2 sec averaging time in a 1 mm stoppered quartz cuvette as reported previously [16,36,47]. The resulting spectra were analyzed using the CONTINLL algorithm and SP43 database employing the CDPRO software package [48,49]. Thermal denaturation experiments were monitored at 222 nm over a temperature range of 4–90°C collecting data at 1°C intervals with a 5 s averaging time. Given that DHDPS requires chaperones to fold [50], the denaturation of DHDPS enzymes is irreversible *in vitro*, and therefore the apparent melting temperature (*T*_M_^*app*^), or midpoint of the transition between folded to unfolded state, was determined empirically from the ordinate maximum of the first derivative of the thermal denaturation profile [36].

### Analytical ultracentrifugation

Analytical ultracentrifugation (AUC) studies were conducted in a XL-I analytical ultracentrifuge (Beckman Coulter) using 12 mm double sector cells with quartz windows loaded into either an An-60 Ti 4-hole rotor or An-50 Ti 8-hole rotor at a temperature of 20°C. *Sp*-DHDPS samples for all centrifugation runs were solubilized in 20 mM Tris-HCl pH 8.0 and 150 mM NaCl. For sedimentation velocity experiments, 380 µL of sample and 400 µL of reference (20 mM Tris-HCl pH 8.0 and 150 mM NaCl) were employed and absorbance *versus* radial profiles were generated at 40,000 rpm in continuous mode using a step size of 0.003 cm and a radial range of 5.8-7.3 cm without averaging. Data were collected at wavelengths of 210 nm (296-740 nM *Sp*-DHDPS) or 230 nm (4.5 µM *Sp*-DHDPS). The absorbance versus radii profiles at different time points were fitted to single discrete species or a continuous size-distribution model using the program SEDFIT [51,52] (available from www.analyticalultracentrifugation.com). The program SEDNTRP [53,54] was used to determine the partial specific volume of *Sp*-DHDPS (0.7475 ml g^-1^), buffer density (1.005 g ml^-1^) and buffer viscosity (1.021 cp) at 20°C. For sedimentation equilibrium experiments, 120 µL of reference solution and 100 µL of sample at three initial protein concentrations (i.e. 296 nM, 355 nM and 740 nM) were centrifuged at rotor speeds of 10,000 rpm and 18,000 rpm. Initial absorbance *versus* radial profiles were measured at 210 nm between 6.8 and 7.2 cm in step mode using a 0.001 cm step size and 3 averages until sedimentation equilibrium was attained (*t* ~ 24 hours). At sedimentation equilibrium, detailed absorbance versus radius scans were taken using a step size of 0.001 cm over a radial range of 6.8 to 7.2 cm with 15 replicates. The resulting absorbance *versus* radial position profiles at multiple sample concentrations and rotor speeds were globally fitted to a single discrete species model or various self-associating models (including monomer-dimer, monomer-trimer, dimer-tetramer, trimer-hexamer and monomer-dimer-tetramer models) using the program SEDPHAT [55] (available from www.analyticalultracentrifugation.com).

### Dynamic light scattering

Dynamic light scattering measurements were made on an ALV 5022F DLS (ALV, Germany). Protein samples were analyzed at a final concentration of 59 μM. Measurements were taken over a 30 s time period with 10 replicates at 20°C. The samples were illuminated with a HeNe laser (633 nm) and experiments were conducted at a scattering angle of 90 degrees. The in-built ALV analysis software was used to determine average radii.

### Crystallization

Crystallization of *Sp*-DHDPS was performed as previously described [40]. Briefly, crystals were obtained by employing the hanging-drop vapor-diffusion method. 1 µl protein solution (10 mg ml^-1^) and 1 µl precipitant solution were equilibrated against 1 ml reservoir solution [0.2 *M* ammonium chloride, 20% (w/v) PEG 6000, 0.1 M MES pH 6.0] in 24-well Linbro plates at 20°C. Crystals were soaked briefly in cryoprotectant composed of 0.2 *M* ammonium chloride, 20% (w/v) PEG 6000, 0.1 M MES pH 6.0 and 20%(w/v) glycerol and were then flash-frozen using liquid nitrogen.

### Structure determination

X-ray diffraction experiments were carried out at the Australian Synchrotron, Victoria, Australia on the MX1 beamline using Blu-Ice [56]. A 1.9 Å resolution data set was integrated and merged with HKL2000 [57] and scaled with SCALA [58]. The crystals were initially assigned to the tetragonal crystal system, space group *P*4_2_2_1_2 with unit cell dimensions *a* = *b* = 105.5 Å and *c* = 62.4 Å, and an overall *R*_merge_ of 6%. Molecular replacement was carried out using the program PHASER [59] with the structure of dihydrodipicolinate synthase from *B. anthracis* (PDB ID: 1XL9) [22] as the search model. One molecule was located in the asymmetric unit. The structure was refined using the program PHENIX [60] with rigid body refinement, simulated annealing and atomic displacement parameters (ADP) refinement. Iterative model building was carried out using the program COOT [61] followed by ADP refinement. Even though the 2*F*_*o*_ *-F*_*c*_ electron density map was clear, there were spurious streaks in the *F*_*o*_ *-F*_*c*_ difference maps and refinement stalled with a *R*_work_ of 24 % and a *R*_free_ of 32 %. Examination of the cumulative intensity plot from SCALA suggested that the data were affected by crystal twinning. The data were inspected further by running the program phenix.xtriage. Since there are no twin laws possible in the above crystal symmetry, over-merging of pseudo-symmetric or twinned data may have been present. The diffraction data were re-integrated in space group P1 using XDS [62] and further analyzed with phenix.xtriage. The presence of seven pseudo-merohedral twin operators were found, two 4-fold and five 2-fold. The twin law giving the lowest *R*_obs_ was -*h*,*l*,*k* superimposing on *h*,*k*,*l*. The data were scaled in space group P2 (cell dimensions *a* = 64.4 Å, *b* = 105.3, Å, *c* = 105.5 Å, β = 90.0° degrees) and refined as above with the inclusion of the above two-fold twin law and four crystallographically-independent subunits (residues 2-299 and 2-297) in the asymmetric unit leading to values for *R*_work_ of 19.7 % and *R*_free_ of 22.9 % (this model has been deposited with PDB ID: 3H5D). However, inspection of the diffraction data and the structural model provided clear evidence for 2_1_ screw axes perpendicular to the monoclinic unique *b* axis. The twinning axis is parallel to the crystallographic two-fold axis (which is a pseudo-4_2_ or 2_1_ screw axis with significant intensity violations), leading to reflections −*k*,*h*,*l* being overlapped with *h*,*k*,*l* and to the observed pseudo-tetragonal 4/mmm Laue symmetry. The final model, now in space group *P*22_1_2_1,_ was solved by molecular replacement using MOLREP [63] with the model PDB ID: 3HIJ [36] together with amplitude-based twin refinement with NCS restraints using REFMAC5 [64]. The final model (*R*_work_ = 16.5% and *R*_free_ = 21.2%) comprises of two monomers (residues A2 – A299 and B2 – B297, and A302 – A311 and B302 – B311), 235 water molecules, 4 potassium ions, 4 glycerol molecules and 2 MES ions. The pseudo-tetragonal symmetry leads to two possible choices of asymmetric unit, one comprising a pair of subunits forming the ‘weak’ dimer interface, the other comprising a pair of subunits forming the ‘tight’ interface – the usual asymmetric unit seen in other tetrameric DHDPS structures. Both were tested, the latter being confirmed as correct. The crystallographic two-fold axis generates the tetramer. Residues 300-301 of chain A and residues 298-301 of chain B were not observed in electron density maps, and as a result of crystal packing, it is not unequivocally clear whether the C-terminal residues 302-311, which were very clearly defined in electron density maps once the twinning and space group problems were solved, indulge in domain swapping to a neighboring tetramer or remain with their own tetramer, However, electrospray ionization mass spectrometry analysis of dissolved crystals of recombinant *Sp*-DHDPS indicates that the entire protein (residues 1-311) is intact. A summary of the crystallographic data collection and refinement statistics is provided in Table 2.

**Table 2:** Data collection and refinement statistics for the X-ray structure of *Sp*-DHDPS (PDB ID: 3VFL).

Temperature (K)			100
Space group			*P22_1_2_1_*
Cell dimensions Å (a, b, c)			62.4, 105.3, 105.5
Resolution (Å)			74.6 -1.9
No. of observations			757223
No. of unique observations			51243
Completeness (%)			99.6 (97.7)^1^
I/σ_I_			35.7 (4.7)
*R*_sym_ (%)**^*2*^**			6.3 (56.2)
Twin operator			-h, l, k
Twin fraction			0.46
Wilson B factor Å^2^			22.6
**REFINEMENT STATISTICS**			
**Non-hydrogen atoms**			
Protein			4725
Water			235
Ligands			52
*R*_work_ (%)**^*3*^**			16.5
*R*_free_ (%)**^*4*^**			21.2
**RMSD values from ideal value**			
Bond lengths (Å)			0.02
Bond angles (°)			2.4
**Ramachandran plot**			
Most favored and allowed region (%)			99.3
***B* factors (Å^2^)**			
Average main chain			27.8
Average side chain			29.6
Average water molecule			29.9

***^1^*** Values in parentheses represent the highest resolution shell (2.1–1.9).

***^2^*** *R*_sym_ = Σ|*I* - (I)|/Σ*I*.

***^3^*** *R*_work_ = Σ||*F*_o_| - |*F*_c_||/Σ|*F*_o_|.

***^4^*** *R*_free_ is based on 5% of the total reflections excluded from refinement.

### Coordinates

Coordinates and structure factors for the final model are accessible *via* the Protein Data Bank (PDB ID: 3VFL).

## Results

### *S. pneumoniae* 447A ΔdapA mutant requires (S)-lysine for growth

**Figure 2:**
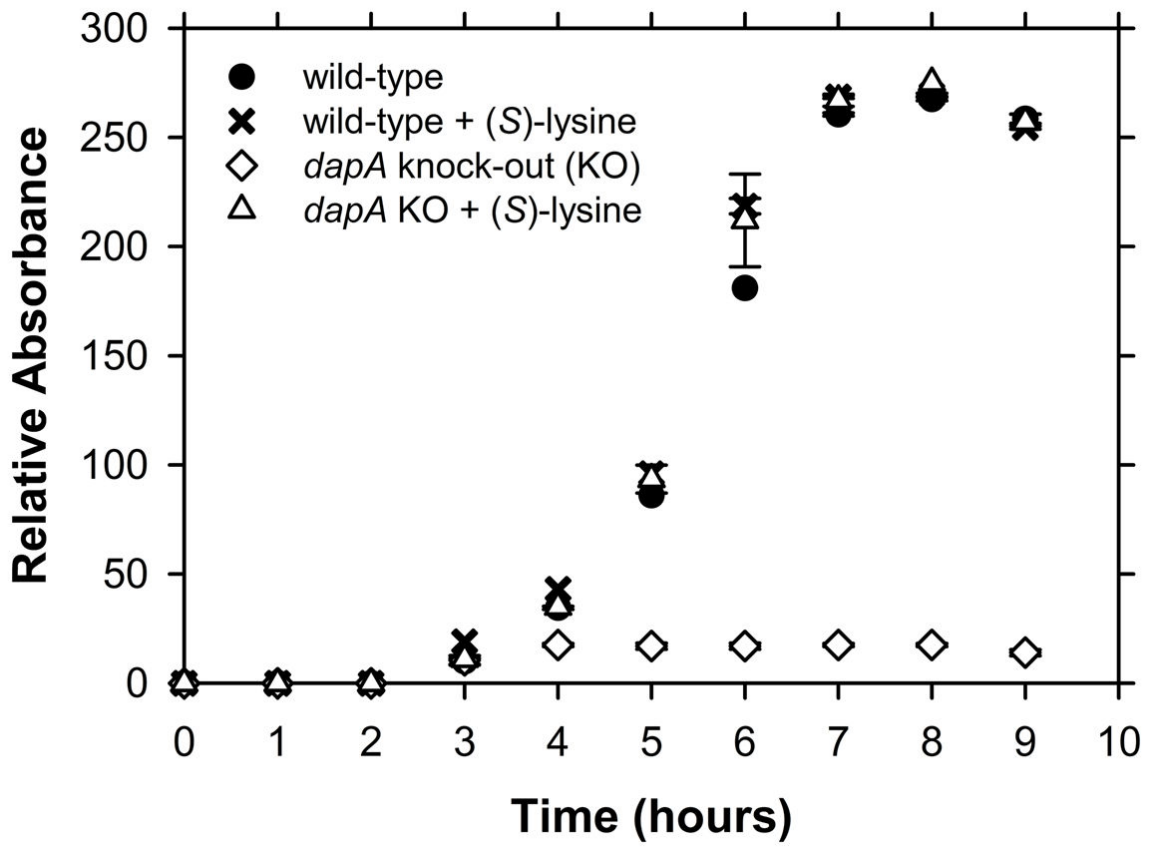
Growth phenotype of the *dapA* knockout strain of *S. pneumoniae*. Growth of wt *S. pneumoniae* 447A (control) and the *ΔdapA* mutant strain in the presence and absence of 20 mM (*S*)-lysine. Growth was assessed by monitoring the optical density (Relative Absorbance) of duplicate cultures on an hourly basis as described in the Materials and Methods.

To validate DHDPS as a promising drug target in *Streptococcus pneumoniae*, the gene encoding this enzyme (i.e. *dapA*) was deleted from strain 447A. The *ΔdapA* mutant was generated by homologous recombination as described in the Materials and Methods. Cultivation of the *ΔdapA* and wild-type strains on nutrient-rich media, such as HBA, showed that their size and morphology were indistinguishable (data not shown). To determine whether the *ΔdapA* mutant required (*S*)-lysine for growth, wild-type and mutant strains were grown in Chemically Defined Medium CDM^+^ and CDM^-^ (where + and - indicates the presence and absence of 200 µg ml^-1^ (*S*)-lysine, respectively) at 37°C in an atmosphere of 5% CO_2_. Analysis of growth rates of wild-type and *ΔdapA* mutant strains showed that the rate of growth of the mutant in CDM^+^ was not significantly different to that of the wild-type strain (Figure 2). However, the *ΔdapA* mutant was unable to grow in CDM^-^ whereas the wild-type strain reached comparable cell density in CDM^+^ and CDM^-^ (Figure 2). This demonstrates that the *dapA* gene, encoding DHDPS, is essential for the growth of *S. pneumoniae* in the absence of lysine.

### Secondary structure and stability of *Sp*-DHDPS

**Figure 3:**
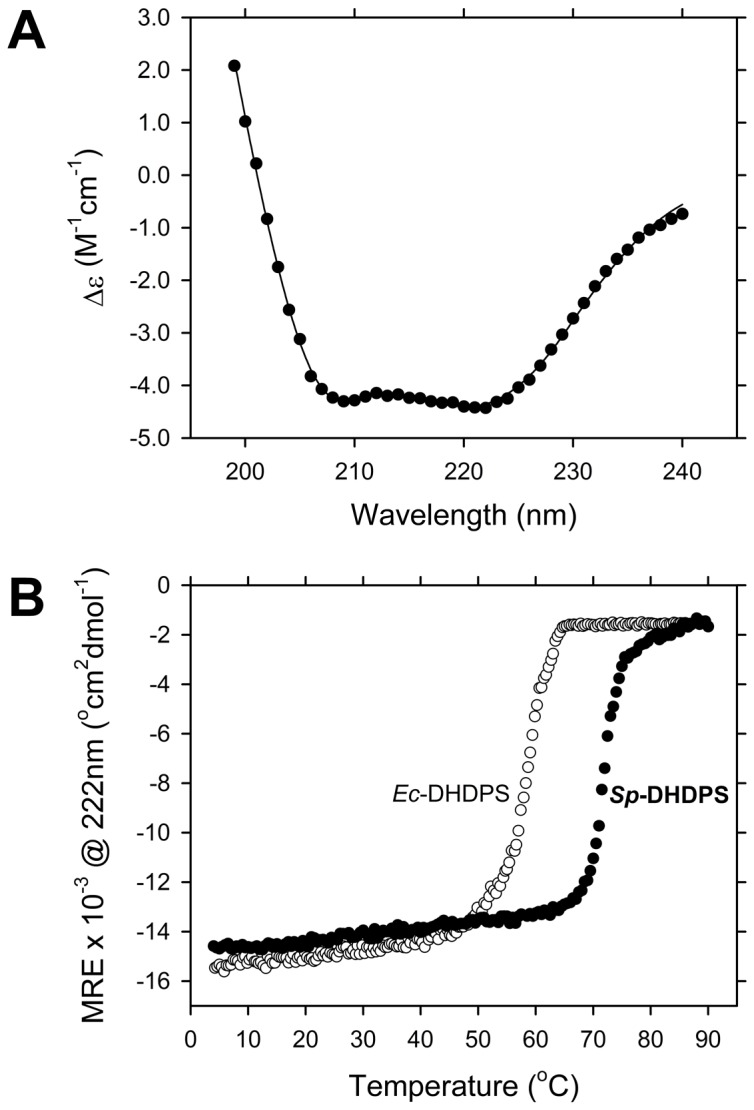
Circular dichroism spectroscopy of *Sp*-DHDPS. (A) CD spectrum of *Sp*-DHDPS () plotted as the molar circular dichroism (Δε) as a function of wavelength. The solid line represents the nonlinear least squares fit using the CONTINLL algorithm and SP43 database within the CDPRO software suite [48.49]. The best-fit resulted in the secondary structure composition reported in Table 3. (B) Thermal denaturation profiles of *Sp*-DHDPS () and *Ec*-DHDPS () plotted as mean residue ellipticity (MRE) versus temperature. The apparent melting temperature (*T*_M_^*app*^), or midpoint of the transition between folded and unfolded states, was determined from the ordinate maximum of a plot of the first derivative of the MRE as a function of temperature to yield 59°C for *Ec*-DHDPS and 72°C for *Sp*-DHDPS.

*Sp*-DHDPS was expressed and purified to >95% homogeneity as described previously [43]. The enzyme was subjected to circular dichroism (CD) spectroscopy in aqueous solution to assess the secondary structure of the recombinant product. The CD spectrum (Figure 3A) shows a broad minimum spanning 208 nm to 222 nm, suggesting *Sp*-DHDPS adopts a mixed α/β secondary structure in solution [16,36,47]. This assertion was confirmed by fitting the CD spectrum of *Sp*-DHDPS to the CONTINLL algorithm and SP43 database using the CDPRO software suite [48,49]. The nonlinear best-fit demonstrates a significant proportion of α-helix and β-strand (Table 3). The calculated secondary structure composition of *Sp*-DHDPS is comparable to that of DHDPS enzymes from other bacteria, including *E. coli* (Table 3). Next we assessed the stability of recombinant *Sp*-DHDPS in solution by conducting thermal denaturation experiments monitored by CD at 222 nm (Figure 3B). The resulting thermal denaturation profile reveals that the enzyme unfolds *via* a single transition with an apparent melting temperature (*T*_M_^*app*^) of 72°C (Figure 3B and Table 4). Recombinant DHDPS from *E. coli* (*Ec*-DHDPS) also unfolds *via* a single transition, but with a significantly lower *T*_M_^*app*^ of 59°C (Figure 3B and Table 4). These data therefore indicate that *Sp*-DHDPS is markedly more stable in solution than *Ec*-DHDPS. We were thus interested in unraveling the molecular mechanism for this enhanced thermostability by assessing the quaternary structure of *Sp*-DHDPS in solution.

**Table 3:** Secondary structure composition of *Sp*-DHDPS and *Ec*-DHDPS determined by CD spectroscopy^***1***^.

**Enzyme**	**α-helix**	**β-strand**	**turn**	**unordered**	**RMSD**
***Sp*-DHDPS**	36	15	20	29	0.05
***Ec*-DHDPS**	34	17	20	29	0.04

^***1***^ Parameters resulting from the nonlinear best-fit using the CONTINLL algorithm and SP43 database within the CDPRO software suite [48,49].

**Table 4:** Comparison of the thermostability and hydrodynamic properties of *Sp*-DHDPS and *Ec*-DHDPS derived from circular dichroism spectroscopy and analytical ultracentrifugation studies.

**Enzyme**	***T*_M_^app1^ °C**	***s*_20,w_ (S)**	***M*_r_^4^ (kDa)**	***M* (kDa)**	***f*/*f*_*0*_^*6*^**	***a/b^7^***	**K_*D*_^42^ (nM)**
***Sp*-DHDPS**	72	7.2^2^	135	133^5^	1.25^6^	2.2	1.7^8^
***Ec*-DHDPS**	59	6.9^3^	125	128^3^	1.26^3^	2.6	76^3^

***^1^*** The apparent melting temperature (*T*_M_^*app*^), or midpoint of the transition between folded and unfolded states, was determined from the ordinate maximum of a plot of the first derivative of the MRE as a function of temperature.

**Figure 4:**
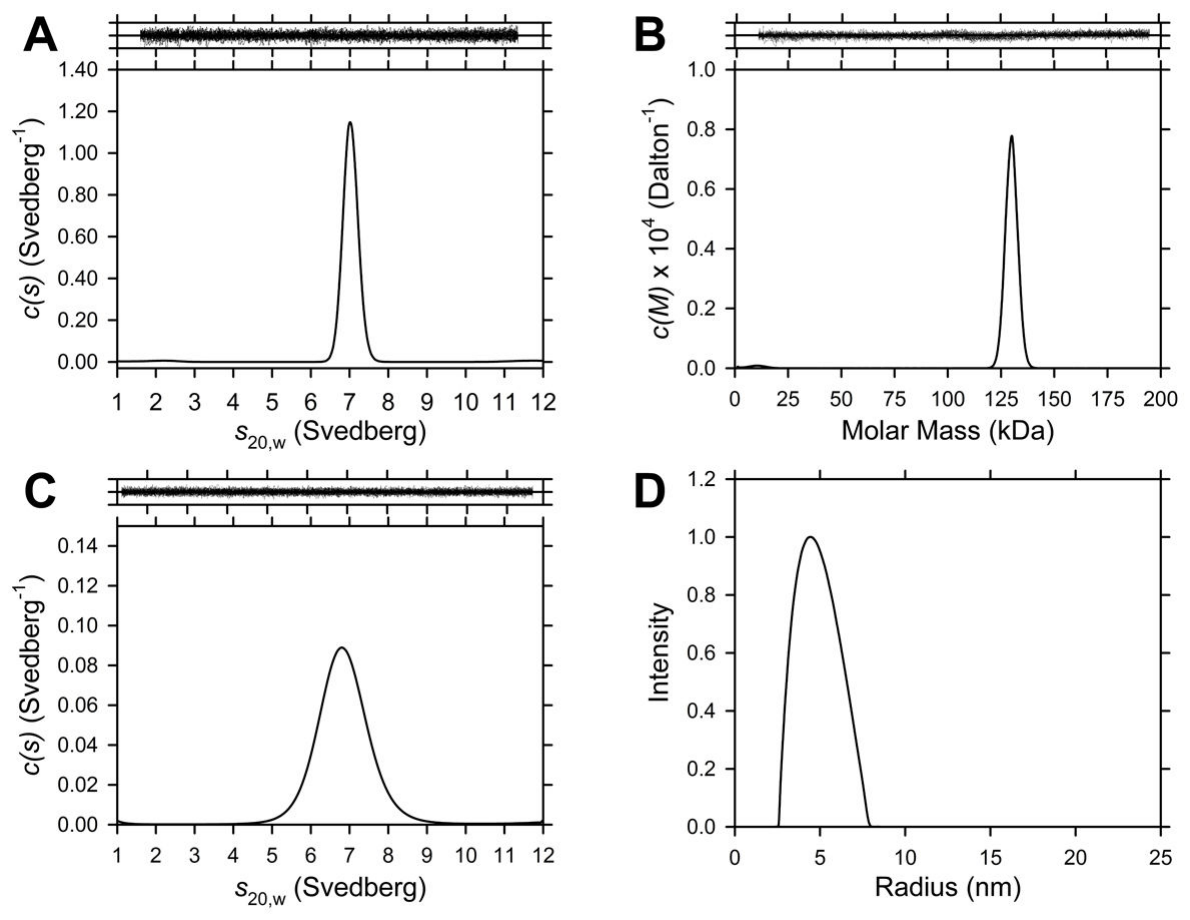
Sedimentation velocity and dynamic light scattering analyses of *Sp*-DHDPS. (A) Continuous sedimentation coefficient, *c*(*s*), distribution of *Sp*-DHDPS at 4.5 µM plotted as a function of standardized sedimentation coefficient (*s*_20,w_) with RMSD and runs test *Z* values for the best-fit of 0.008 and 6.6, respectively. (B) Continuous mass, *c*(*M*), distribution of *Sp*-DHDPS at a concentration of 4.5 µM plotted as a function of molar mass with RMSD and runs test *Z* values of 0.008 and 6.6 respectively. (C) *c*(*s*) distribution of *Sp*-DHDPS at 740 nM, with RMSD and runs test *Z* values of 0.02 and 1.64, respectively. Data were analyzed employing the program SEDFIT [51,52,65,66] using a resolution (N) of 200 and a sedimentation coefficient range of 0.1-12 S or molar mass range 1.0-250 kDa with a P-value of 0.95. *Above* *Panels* *A-C*: Residuals plotted as a function of radial position resulting from continuous size-distribution best-fits shown in panels A-C. (D) A distribution plot of the average unweighted hydrodynamic radii of *Sp*-DHDPS at a concentration of 59 µM determined using dynamic light scattering (DLS). The results of the distribution plot reveal an average hydrodynamic radius of 4.5 ± 0.2 nm.

***^2^*** Value determined experimentally from the ordinate maximum of the *c*(*s*) distribution best-fit shown in Figure 4A.

***^3^*** Hydrodynamic properties reported in [34].

***^4^*** Molecular mass calculated from the amino acid sequence.

***^5^*** Value calculated experimentally from the apparent molecular mass taken from the ordinate maximum of the *c*(*M*) distribution best-fit shown in Figure 4B.

***^6^*** Frictional ratio calculated using the partial specific volume () method employing SEDNTERP software [53,54].

***^7^*** Axial-ratio as calculated from the program SEDNTERP using the method assuming a prolate ellipsoid.

**Figure 5:**
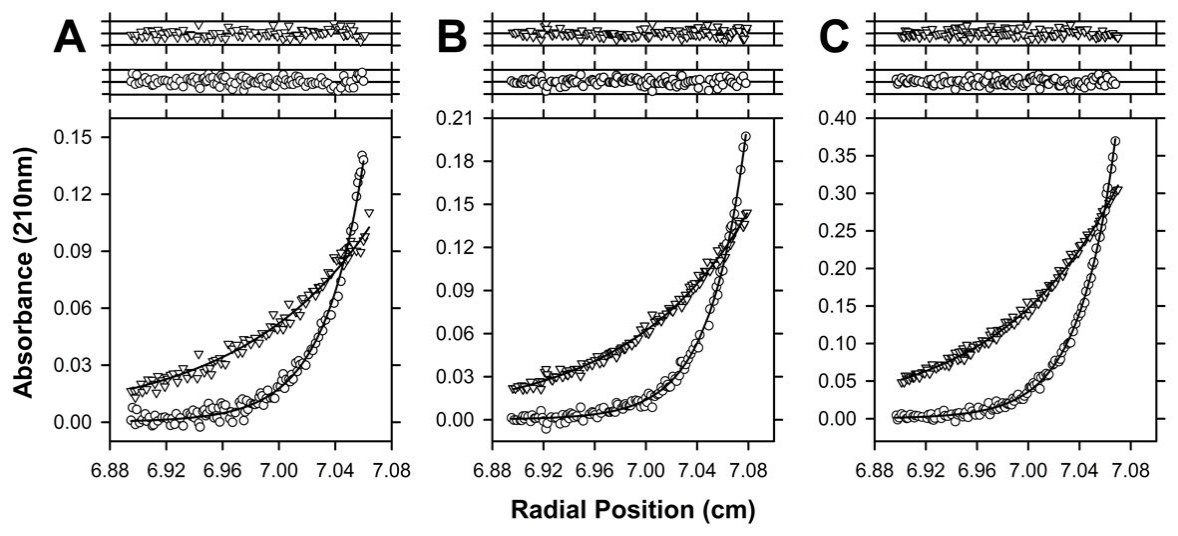
Sedimentation equilibrium analyses of *Sp*-DHDPS. Absorbance at sedimentation equilibrium is plotted as a function of radial position for *Sp*-DHDPS at an initial concentration of (A) 296 nM; (B) 355 nM and (C) 740 nM. Data was measured at 210 nm and rotor speeds of 10,000 rpm () and 18,000 rpm (). The resulting global nonlinear least squares fit to a dimer-tetramer equilibrium model is shown as solid lines and yielded a K_D_^4à2^ of 1.7 nM with a global reduced χ^2^ of 0.5. *Above* *Panels* *A-C*: Residuals plotted as a function of radial position resulting from the global nonlinear best-fit analysis to a dimer-tetramer model for data at 10,000 rpm () and 18,000 rpm ().

***^8^*** The tetramer-dimer dissociation constant calculated from the global nonlinear least squares best-fit described in Figure 5

### Quaternary structure of *Sp*-DHDPS in aqueous solution

To gain further insights into the solution stability of *Sp*-DHDPS, sedimentation velocity studies were conducted in the analytical ultracentrifuge. Absorbance *versus* radial data profiles at different time points were fitted to a continuous size-distribution, *c*(*s*), model [51.52,65,66]. The resulting *c*(*s*) distribution for *Sp*-DHDPS at an initial concentration of 4.5 µM is shown in Figure 4A. The *c*(*s*) distribution reveals a single peak with a *s*_20,w_ value of 7.2 S that is consistent with a tetrameric species (Table 4) [34,36]. The corresponding *c*(*M*) distribution (Figure 4B) confirmed this assertion and shows a single peak with a molar mass of 133 kDa, which closely matches the theoretical mass of the *Sp*-DHDPS tetramer (135 kDa). Sedimentation studies at a 6-fold lower protein concentration (i.e. 740 nM) were conducted and the resulting *c*(*s*) nonlinear least-squares fit also reveals the presence of a single peak (Figure 4B) with a *s*_20,w_ value of 7.0 S, consistent with the enzyme existing primarily as a tetramer [26,27,34,36]. These sedimentation velocity analyses suggest that *Sp*-DHDPS exists as a very stable tetramer in solution. Sedimentation equilibrium experiments were subsequently performed in the analytical ultracentrifuge to examine the strength of subunit interactions and the resulting data at multiple protein concentrations fitted to various equilibrium schemes. Not surprisingly, the optimal global nonlinear least-squares fit was obtained for a dimer-tetramer equilibrium model (Figure 5, solid lines) with a tetramer-dimer dissociation constant (*K*_D_^42^) of 1.7 nM. Interestingly, the calculated *K*_D_^42^ for *Sp*-DHDPS is considerably tighter than that obtained for the previously characterized *Ec*-DHDPS tetramer (Table 4) [34]. We next set out to determine the average hydrodynamic radius of the *Sp*-DHDPS tetramer in solution using dynamic light scattering.

### The hydrodynamic radius of the *Sp*-DHDPS tetramer

The quaternary structure and hydrodynamic radius of *Sp*-DHDPS were studied by dynamic light scattering (DLS) experiments at an enzyme concentration of 59 μM [3.5 × 10^4^-fold above the *K*_D_^4→2^ of *Sp*-DHDPS (Table 4)]. The resulting data were analyzed by the method of cumulants (second-order) and were found to be reproducible between runs and independent of the choice of fit range. Figure 4D shows a regularized distribution fit employing the in-built software (ALV). Analysis of data resulted in a range of hydrodynamic radii that centered on an average value of 4.5 ± 0.2 nm. The average hydrodynamic radius was consistent between runs and was not affected by the choice of fit parameters (correlation function limits or radius limits). By comparison, the Stokes radius of the tetramer calculated from sedimentation velocity studies is 4.3 nm, which is in excellent agreement with the DLS experiment.

### Enzyme kinetic properties of *Sp*-DHDPS

**Figure 6:**
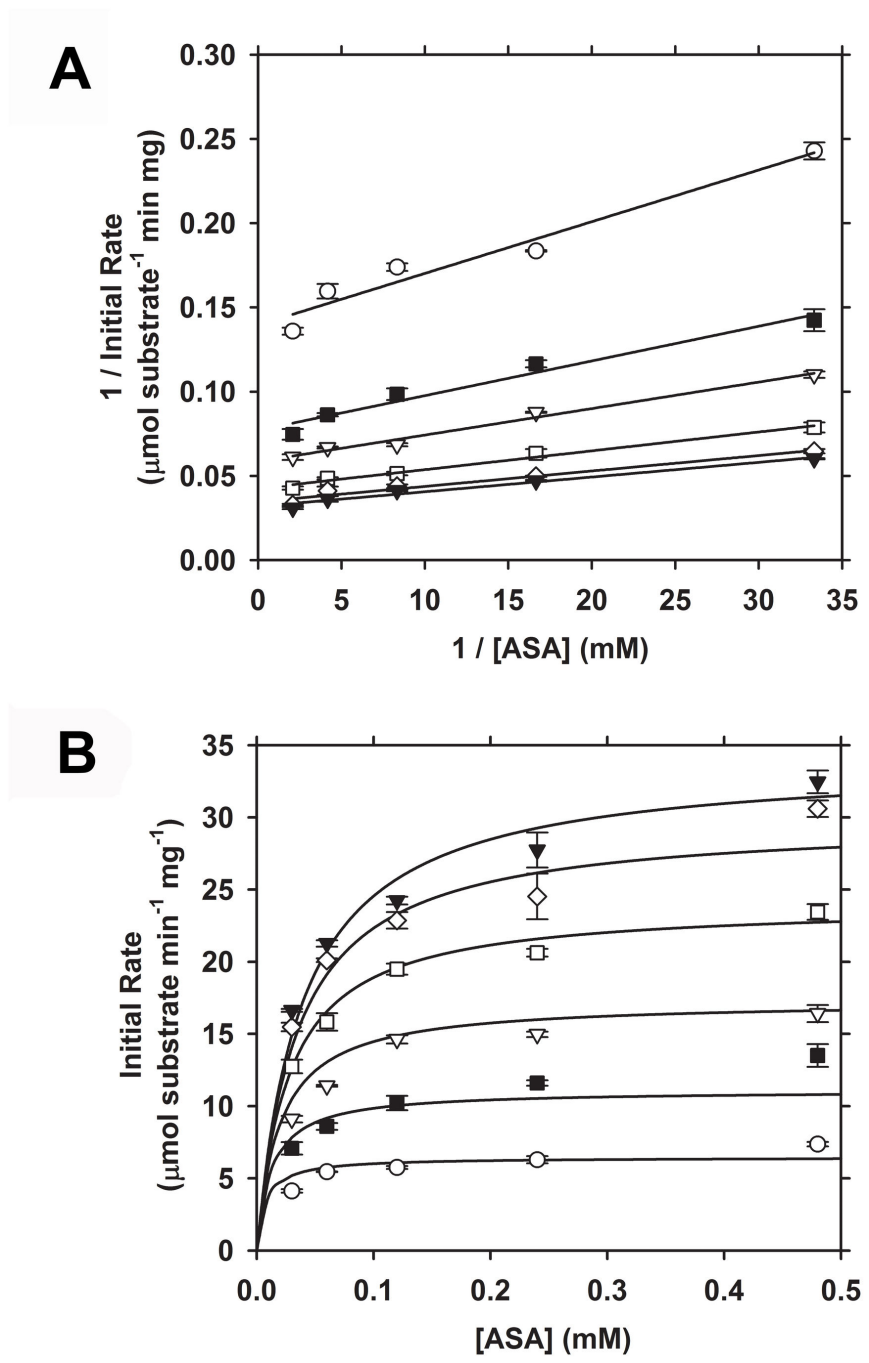
Enzyme kinetic profiles of recombinant *Sp*-DHDPS. Initial velocity was measured as a function of (*S*)-ASA concentration. Experiments were conducted at fixed pyruvate concentrations of () 0. 5 mM, () 1.0 mM, () 2.0 mM, () 4.0 mM, () 8.0 mM and () 16.0 mM. (A) Lineweaver-Burk plots showing multiple parallel lines; diagnostic of a ping pong kinetic mechanism [25,67,69]. (B) Michaelis-Menten plots of data shown in A, where solid lines represent the global nonlinear best-fit to a Ping-Pong mechanism (without substrate inhibition) using ENZFITTER software, resulting in a *R* ^2^ = 0.98 and the enzyme kinetic parameters summarized in Table 5.

To characterize the catalytic properties of the *Sp*-DHDPS tetramer, we employed the DHDPS-DHDPR coupled assay [45] at an enzyme concentration of 20 nM, which is ~12-fold greater than the *K*_D_^4→2^ (Table 4). Initial rates were measured with fixed pyruvate concentrations of 0.5 mM, 1.0 mM, 2.0 mM, 4.0 mM, 8.0 mM and 16.0 mM and varying ASA concentrations (0-0.48 mM). The resulting data were expressed initially as Lineweaver-Burk plots (Figure 6A), which displayed a characteristic series of parallel lines indicating that *Sp*-DHDPS follows a Ping-Pong kinetic mechanism [67]. This mechanism has been demonstrated for DHDPS orthologs from other bacterial species, including *Ec*-DHDPS [45,68,69]. The data were subsequently plotted to produce Michaelis-Menten profiles (Figure 6B) and fitted to bi-substrate kinetic models (with and without substrate inhibition), namely the ternary complex and Ping-Pong mechanism employing ENZFITTER software (Biosoft). The global nonlinear regression analysis yielded a best-fit to a Ping-Pong model with no substrate inhibition (Figure 6B, solid lines) that resulted in a *R* ^2^ = 0.98 and the kinetic parameters reported in Table 5. The *K*_M_ of *Sp*-DHDPS for (*S*)-ASA is similar that for the *E. coli* enzyme (Table 5). However, the *K*_M_ for pyruvate is 10-fold higher than that for *Ec*-DHDPS, and the catalytic turnover (*k*_cat_) is 6-fold lower for *Sp*-DHDPS compared to the *E. coli* ortholog (Table 5). 

**Table 5:** Summary of the enzyme kinetic parameters of *Sp*-DHDPS compared to *Ec*-DHDPS.

**Enzyme**	***k*_*cat*_ (sec^-1^)**	***K*_M_^*ASA*^ (m*M*)**	***K*_M_^*PYR*^ (m*M*)**
***Sp*-DHDPS**	22	0.044 ± 0.003	2.55 ± 0.05
***Ec*-DHDPS^*1*^**	124	0.11 ± 0.01	0.26 ± 0.03

***^1^*** Kinetic parameters reported in [25].

### Crystal structure of *Sp*-DHDPS

**Figure 7:**
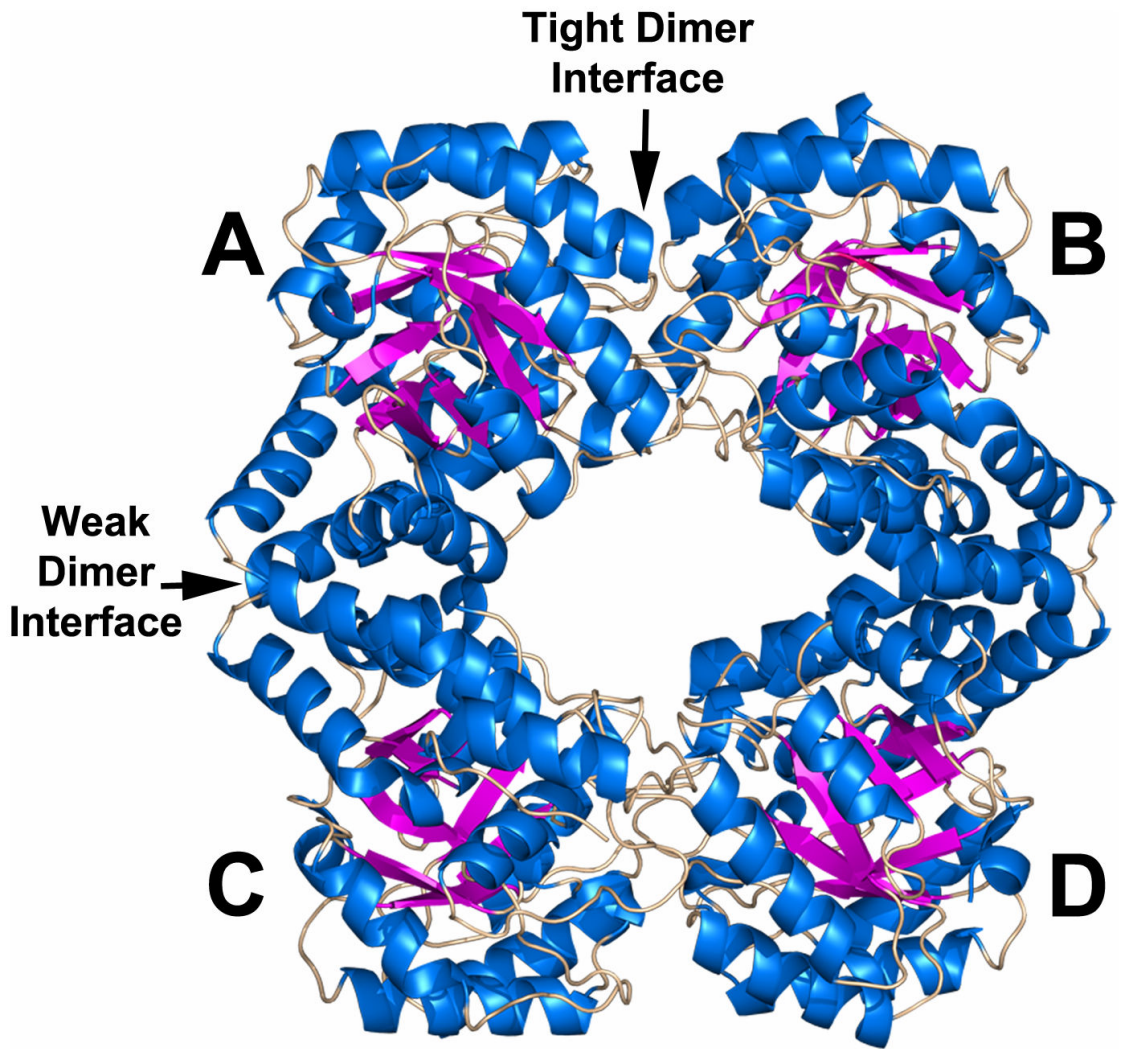
Crystal structure of *Sp*-DHDPS determined at a resolution of 1.9 Å. The enzyme crystallizes as a tetramer comprised of four identical subunits labeled A, B, C & D with subunits AB or CD connected *via* the ‘tight’ dimer interface, and subunits AC or BD connected *via* the ‘weak’ dimer interface.

In order to gain further insight into the stability and kinetic behavior at the atomic level, we next sought to determine the crystal structure of *Sp*-DHDPS. The collection of high-resolution synchrotron X-ray data has been reported recently [43]. Crystals were pseudo-merohedrally twinned to give the illusion of tetragonal symmetry, with a twin fraction of 0.46 (Table 2). Moreover, the apparent 4_2_ screw axis was characterized by significant systematic absence violations, which led after abortive attempts in *P*4_2_2_1_2 to solution and refinement in the monoclinic space group *P*2_1_ where there is ambiguity with respect to the twin law. However, with two well-defined 2_1_ screw axes perpendicular to the pseudo-tetragonal axis, final refinements proceeded successfully in the orthorhombic space group *P*22_1_2_1_ with the twinning axis being parallel to the pseudo-tetrad. Only in this final assignment of crystallographic symmetry did the C-terminal residues 302-311 (see below) become well defined. The structure has been refined at 1.9 Å resolution (Table 2) to an *R*_work_ of 16.5 % (*R*_free_ of 21.2 %). Two residues, V147 and Y114 in both chains A and B lie in disallowed Ramachandran conformers, but are clearly defined in the electron density. Both residues are located at the ‘tight’ dimer interface (Figure 7) and are held in strained conformations by hydrogen bonding and hydrophobic packing at this interface. The strained conformation of the highly conserved Y114 (Figure 1B) has been observed in all DHDPS structures determined to date, including those from plants [20-36]. However, the strained conformation of V147, which is poorly conserved (Figure 1B), appears to be unique to *Sp*-DHDPS.

**Figure 8:**
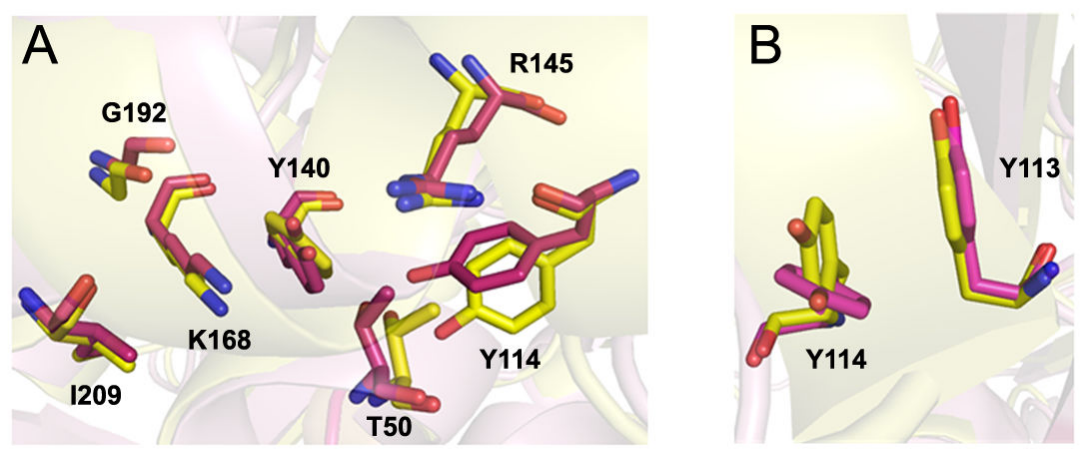
Comparison of the active sites of *Sp*-DHDPS (yellow) and *Ec*-DHDPS (pink). (A) An overlay of the active site with residues labeled according to *Sp*-numbering. (B) Image demonstrating the approximate 70° rotation of Y114 in the active site of *Sp*-DHDPS compared to the equivalent residue in *Ec*-DHDPS (Y107).

Consistent with the solution studies, *Sp*-DHDPS forms a homotetrameric structure (PDB ID: 3VFL) that is depicted in Figure 7. Each monomer unit is folded to form an N-terminal (β/α)_8_-barrel (residues 2-229) with a C-terminal extension comprised of three helices (residues 232-292). The C-terminal residues 302-311 lie in a groove formed by the ‘tight’ dimer interface (i.e. the interface between chains A & B or C & D in Figure 7). As with other DHDPS structures, including *E. coli* DHDPS [25,31], the active site of *Sp*-DHDPS is located within the β-barrel of each subunit. Many of the residues adopt a similar spatial arrangement to that of the *Ec*-DHDPS structure [PDB ID: 1YXC] [25] (Figure 8A). The key lysine residue, K168, which forms a Schiff base with the pyruvate substrate, is present alongside residues Y140, R145, and G192, which play a pivotal role in the cyclization after reaction with the second substrate (*S*)-ASA [25,70] (Figure 8A). Two of the residues forming the catalytic triad, which serves to shuttle protons to and from the active site (i.e. Y140 and T50), are also present in a similar orientation to the equivalent residues from the *E. coli* ortholog (Figure 8A). However, the third residue of the catalytic triad, Y114, undergoes a ~70° rotation in the active site of *Sp*-DHDPS relative to the equivalent residue of the *E. coli* enzyme (i.e. Y107), although the functionally important –OH group is similarly positioned. This residue interdigitates across the ‘tight’ dimer interface forming a hydrophobic stack with Y113 from the adjacent monomer (Figure 8B). This 70° rotation serves to change the interaction to a π stacking between the two tyrosine residues.

**Figure 9:**
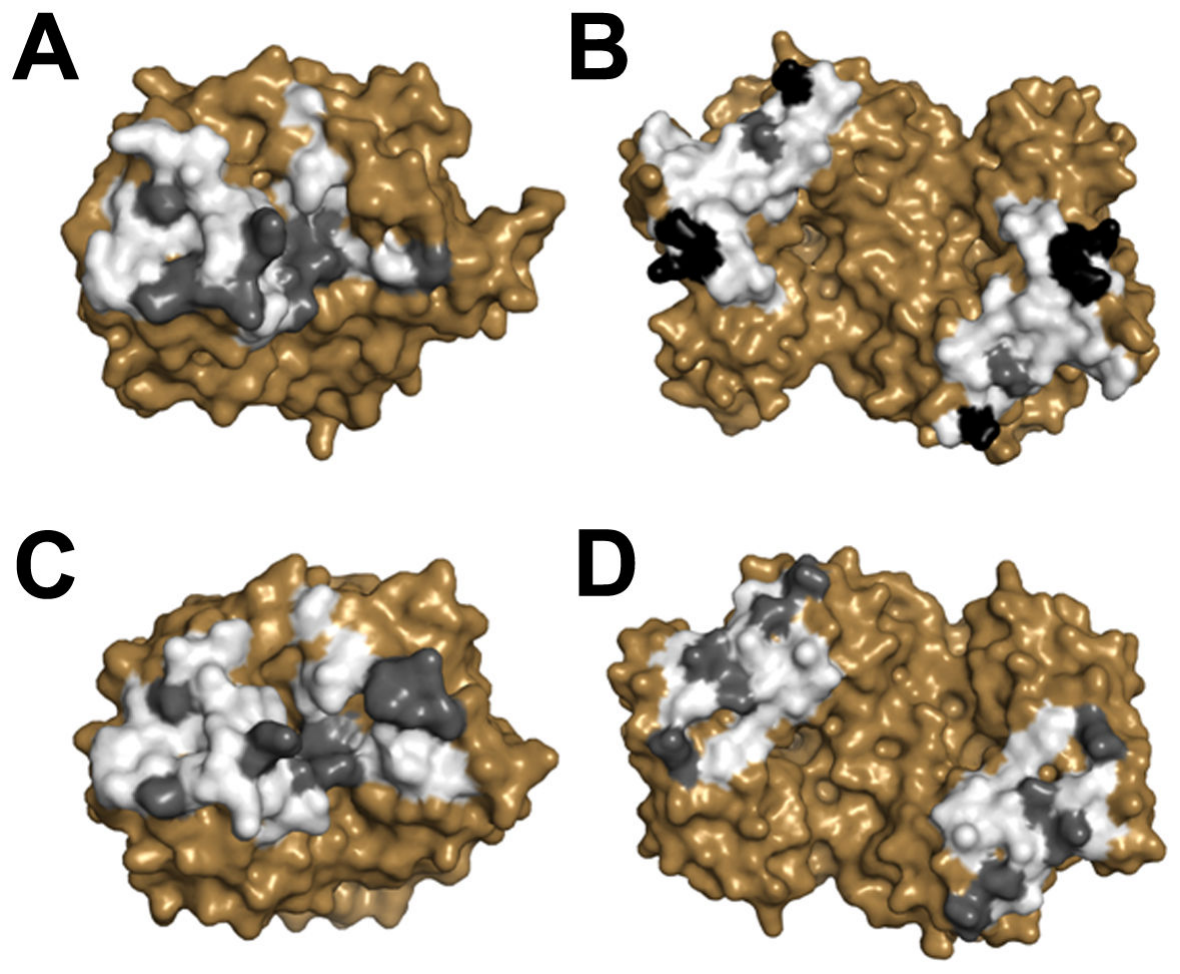
Comparison of the oligomeric interfaces of DHDPS from *S. pneumoniae* (panels A & B) and *E. coli* (panels C & D). Noncovalent interactions and solvent-inaccessible surface area (SISA) were calculated using the program PISA [71]. The ‘tight’ dimer interface of *Sp*-DHDPS (panel A) buries 1350 Å^2^ of SISA compared to *Ec*-DHDPS (panel C), which buries 1290 Å^2^ of SISA. The ‘weak’ dimer interface of *Sp*-DHDPS (panel B) buries 800 Å^2^ of SISA compared to 500 Å^2^ for *Ec*-DHDPS (panel D). White, grey and black shaded regions correspond to interfacing, hydrogen bonding and ion bonding residues, respectively.

Relative to the *Ec*-DHDPS structure (PDB ID: 1YXC) [26], insight into the enhanced stability observed for *Sp*-DHDPS in solution is revealed by close inspection of the interactions stabilizing the ‘tight’ dimer interface, and in particular, the ‘weak’ dimer interface (Figure 7, interface AC or BD). Analysis of the solvent inaccessible surface areas (SISA), calculated using the Protein Interfaces, Surfaces and Assemblies (PISA) program [71], reveals that the ‘tight’ dimer interface of *Sp*-DHDPS (Figure 7, interface AB or CD) buries 1350 Å^2^ (Table 6, Figure 9A), slightly more than that calculated (1290 Å^2^) for the equivalent interface of the *Ec*-DHDPS structure (Table 6, Figure 9C). Although both enzymes contain the same number of residues (i.e. 38 in total) at this interface, *Sp*-DHDPS contains a larger proportion of residues forming hydrogen bonds. In contrast, the ‘weak’ dimer interface has a total SISA of 800 Å^2^ (Table 6, Figure 9B), which is significantly greater than the equivalent interface of the *E. coli* structure (500 Å^2^) (Table 6 & Figure 9D). The substantially larger buried surface area in *Sp*-DHDPS is due to a greater number of residues forming contacts at this interface. In addition, it is interesting to note that this enzyme also contains three residues that form salt bridge interactions, a feature that is absent in *Ec*-DHDPS (Table 6 & Figure 9D). 

**Table 6:** Comparison of the ‘tight’ dimer and ‘weak’ dimer interfaces of *Sp*-DHDPS and *Ec*-DHDPS.

**Enzyme**	**Tight Dimer SISA^*1*^ (Å^2^)**	**Residues involved in Hydrophobic contacts**	**Residues involved in Hydrogen bonding**	**Residues involved in Ion-Ion interactions**	**Weak Dimer SISA^*1*^ (Å^2^)**	**Residues involved in Hydrophobic contacts**	**Residues involved in Hydrogen bonding**	**Residues involved in Ion-Ion interactions**
***Sp*-DHDPS**	1350	27	11	0	800	20	4	3
***Ec*-DHDPS**	1290	31	7	0	500	11	6	0

***^1^*** SISA = solvent inaccessible surface area. Calculated employing PISA [71] analysis (http://www.ebi.ac.uk/msd-srv/prot_int/pistart) and PDB coordinates for *Sp*-DHDPS (PDB ID: 3VFL) and *Ec*-DHDPS (PDB ID: 1YXC).

## Discussion

*Streptococcus pneumoniae* is one of the leading causes of disease in the world. In the United States, for example, the organism is responsible for 500,000 cases of pneumonia, 50,000 cases of bacteremia, and 3,000 cases of meningitis per annum [3,72]. Current treatment relies primarily upon the use of penicillin-based antibiotics. However, this approach has had significant limitations given the emergence of drug-resistant *S. pneumoniae* (DRSP). The appearance of multiple drug resistant (MDR) strains in recent times and the occurrence of community acquired infection are also of particular concern [72,73]. Accordingly, there is an urgent need to develop novel antimicrobials and an equally urgent need to discover new antibiotic targets. A promising pneumococcal drug target is DHDPS, given the enzymes function in producing lysine required for protein and cell wall synthesis. However, our knock-out studies show that the *ΔdapA* strain of *S. pneumoniae* is a lysine auxotroph (Figure 2), which suggests DHDPS attenuated strains may be able to survive *in vivo* by scavenging exogenous lysine from the host. Further studies are required to ascertain the viability of the *ΔdapA* strain *in vivo*. Nevertheless, having established the *ΔdapA* strain was essential to *S. pneumoniae* in minimal media, we set out to characterize the structure, function and stability of the enzyme to afford insight into rational drug design strategies to afford the discovery of *Sp*-DHDPS inhibitors in future work.

Our studies show that *Sp*-DHDPS is similar in some respects to the well characterized ortholog from *E. coli*, which shares 36 % sequence identity. For instance, CD spectroscopy and AUC studies reveals that *Sp*-DHDPS, like *Ec*-DHDPS, has high α/β secondary structure (Table 3, Figure 3A) and resides in a dimer-tetramer equilibrium (Table 4, Figures 4 and 5). Likewise, enzyme kinetic studies demonstrate both *Sp*-DHDPS and *Ec*-DHDPS operate via a Ping-Pong catalytic mechanism. However, the enzyme kinetic parameters calculated for *Sp*-DHDPS are significantly different to the *E. coli* enzyme (Table 5). The greater *K*_M_^*PYR*^ for *Sp*-DHDPS suggests a lower thermodynamic affinity for this substrate (assuming the coupling of pyruvate with (*S*)-ASA is rate-determining), consistent with the very strongly associated, and presumably more rigid tetramer, that hinders access of pyruvate to the binding site and relaxation of the protein to accommodate pyruvate. This is consistent with our prior conclusions that protein dynamics control enzyme kinetics [26,27,36,74]. 

It is worthwhile noting that pyruvate is a central metabolite, serving as a substrate for several other enzymes. Pyruvate-utilizing enzymes, such as pyruvate dehydrogenase and lactate dehydrogenase from Gram-positive bacterial species, including *Bacillus subtilis*, *Corynebacterium glutamicum* and *Lactococcus lactis*, display *K*_M_^*PYR*^ ranging 1 - 15 mM [75,76]. These values are considerably higher than orthologs from Gram-negative species, such as *E. coli* and *Thermotoga maritima*, where *K*_M_^*PYR*^ values range from 0.018 - 0.43 mM [77-79]. These differences, found also in comparison of DHDPS from Gram-positive and Gram-negative species, may indicate that pyruvate is present at a higher intracellular steady-state concentration in *S. pneumoniae* (and other Gram-positive bacteria) than in *E. coli* (and other Gram-negative bacteria). The origins of these differences in *K*_M_^*PYR*^ are not apparent from the structures of these DHDPS enzymes.

Solution studies also showed *Sp*-DHDPS possesses significantly greater thermostability compared to *Ec*-DHDPS (Figure 3B). We subsequently demonstrated using analytical ultracentrifugation that the enhanced thermostability of *Sp*-DHDPS is contributed by a 45-fold tighter tetramer-dimer dissociation constant (Table 4) [34]. We therefore determined the three-dimensional structure of *Sp*-DHDPS using X-ray crystallography (Figure 7) to provide insight into the enhanced thermal and thermodynamic stability. The origin of the enhanced thermal stability is clearly revealed in the 1.9 Å resolution crystal structure of *Sp*-DHDPS (Figure 7) that shows a tetrameric structure of the enzyme, consistent with our solution studies (Table 4, Figures 4 and 5). The tertiary and quaternary structure architecture of *Sp*-DHDPS is very similar to *Ec*-DHDPS [25,31,34] with an overall RMSD for superposition of the tetramers of 1.1 Å (alpha carbon atoms), as well as to other structurally characterized bacterial DHDPS enzymes [16-19,22,24-27,29-36];. However, significant structural differences are observed between *Sp*-DHDPS and *Ec*-DHDPS at the subunit interfaces (Figure 9). We show that there is an increase of 60 Å^2^ and 300 Å^2^ in solvent-inaccessible surface area (SISA) at the ‘tight’ dimer and ‘weak’ dimer interfaces of *Sp*-DHDPS, respectively (Table 6, Figure 9). This significant increase in SISA, in combination with the greater proportion of hydrogen bonding residues at the ‘tight’ dimer interface, as well as the presence of residues participating in salt bridge interactions at the ‘weak’ dimer interface (also referred to as the tetramerization interface [36]), is consistent with the 45-fold lower tetramer-dimer dissociation constant for *Sp*-DHDPS (Table 4, Figure 5), and to its considerably higher thermal stability (Figure 3B). Consistent with previous studies of other DHDPS enzymes, the residues that form interactions at the ‘weak’ dimer interface are poorly conserved in *Sp*-DHDPS, whereas strong conservation is observed at the ‘tight’ dimer interface where the active sites are located [16-36]. Given that recent studies show dimeric mutants of DHDPS have significantly attenuated catalytic function compared to the wild-type tetramers [26,27,36], the poor conservation at the ‘weak’ dimer interface offers potential for the design of pathogen-specific antimicrobial agents [34], particularly given that protein-protein interfaces represent highly specific drug targets [80,81]. Indeed, with the increase in drug-resistant bacteria linked to the overuse and misuse of broad spectrum antibiotics [8,9], exploiting the ‘weak’ dimer interface of *Sp*-DHDPS may provide a means to negate the incidence of broad spectrum drug resistance. 

In conclusion, through gene knock-out studies, circular dichroism spectroscopy, analytical ultracentrifugation, dynamic light scattering, enzyme kinetics and X-ray crystallography studies, we demonstrate that *Sp*-DHDPS is an essential, active and thermostable tetramer. Our work offers insight into rational drug design strategies targeting multiple sites of the enzyme to afford the discovery of novel antibiotic agents with potential to negate drug resistance.
